# Improving Graph Convolutional Network with Learnable Edge Weights and Edge-Node Co-Embedding for Graph Anomaly Detection

**DOI:** 10.3390/s24082591

**Published:** 2024-04-18

**Authors:** Xiao Tan, Jianfeng Yang, Zhengang Zhao, Jinsheng Xiao, Chengwang Li

**Affiliations:** 1 School of Electronic Information, Wuhan University, Wuhan 430072, China; haiantanxiao@whu.edu.cn (X.T.); xiaojs@whu.edu.cn (J.X.); 2 School of Software Engineering, University of Science and Technology of China, Suzhou 215123, China; gavin@ustc.edu.cn; 3 College of Sciences, China Jiliang University, Hangzhou 310018, China; licw980824@126.com

**Keywords:** graph anomaly detection, label propagation, graph convolutional neural networks, semi-supervised learning

## Abstract

The era of Industry 4.0 is gradually transforming our society into a data-driven one, which can help us uncover valuable information from accumulated data, thereby improving the level of social governance. The detection of anomalies, is crucial for maintaining societal trust and fairness, yet it poses significant challenges due to the ubiquity of anomalies and the difficulty in identifying them accurately. This paper aims to enhance the performance of the current Graph Convolutional Network (GCN)-based Graph Anomaly Detection (GAD) algorithm on datasets with extremely low proportions of anomalous labels. This goal is achieved through modifying the GCN network structure and conducting feature extraction, thus fully utilizing three types of information in the graph: node label information, node feature information, and edge information. Firstly, we theoretically demonstrate the relationship between label propagation and feature convolution, indicating that the Label Propagation Algorithm (LPA) can serve as a regularization penalty term for GCN, aiding in training and enabling learnable edge weights, providing a basis for incorporating node label information into GCN networks. Secondly, we introduce a method to aggregate node and edge features, thereby incorporating edge information into GCN networks. Finally, we design different GCN trainable weights for node features and co-embedding features. This design allows different features to be projected into different spaces, greatly enhancing model expressiveness. Experimental results on the DGraph dataset demonstrate superior AUC performance compared to baseline models, highlighting the feasibility and efficacy of the proposed approach in addressing GAD tasks in the scene with extremely low proportions of anomalous data.

## 1. Introduction

In real-world settings, anomalies are both ubiquitous and potentially detrimental, yet challenging to identify. Fraudulent activities pose significant threats to the robust functioning of society, not only in economic terms but also by adversely impacting societal trust and fairness. Anomaly detection, also referred to as fraud detection, is assuming an increasingly crucial role in contemporary society. Its applications range from detecting fake news [1] to identifying fraudulent reviews [2].

In academia, “anomaly” is commonly abstracted as a set of data that deviates from the majority [3]. Despite the development of numerous techniques in recent years for uncovering outliers and anomalies in unstructured sets of multi-dimensional points, the scarcity of anomalous behavior, high labeling costs, and privacy protection policies result in a paucity of data samples labeled as anomalous [3].

We observe that graphs in the current graph anomaly detection domain mainly exhibit the following three characteristics.

1. Nodes are Labeled as well as Featured. Nodes in graphs often possess both label and feature properties. Node labels can propagate and aggregate along the edges of the graph, while node features can propagate along the edges and undergo transformations through neural network layers. Both label and feature propagation are beneficial for predicting node behavior. The effective combination of these two propagation aspects warrants attention.
2. Edges Contain Rich Information. In the field of GAD, the topology and structure of the graph contain a wealth of information. The attributes and directions of edges imply that even in isomorphic graphs, there are multiple types of connections (i.e., edges) between different nodes. For instance, relationships between users may include transfer and recipient relationships, loan and borrower relationships, as well as familial relationships. This information can assist in better classification, thereby identifying anomalous nodes. However, this information is often overlooked by other methods.
3. The Proportion of Anomalous Data is Extremely Low. While fraud incidents do occur, the majority of users in reality are normal users. The proportion of fraudulent users is significantly smaller than that of normal users, making fraud detection a classic example of imbalanced data. In machine learning model training, achieving a relatively balanced distribution of various sample classes is crucial for optimal performance. When there is a substantial difference in the proportions of different classes, the trained model tends to exhibit characteristics more aligned with the majority class, thereby neglecting features associated with minority class samples.

The inherent suitability of graph structures for analyzing relationships and connectivity patterns in networks has propelled GAD to the forefront. GAD abstracts traditional streaming data into graph structures, where nodes represent entities and edges signify relationships between entities. The interconnected nodes form edges that convey structural information about the graph, information that can be stored using an adjacency matrix [4]. GAD aims to detect anomalies by leveraging the structural information inherent in the network, combining it with classic anomaly detection approaches. Considering fraud scenarios, fraudulent activities often exhibit associations and collaboration. The connections between fraud groups are typically closer than those between normal users, and the behaviors demonstrate a certain level of similarity. This also explains why graph analysis can be employed to detect anomalous behaviors in practical scenarios. According to the different basic graph algorithms, GAD algorithms can be classified into several types, including community detection, feature discovery, pattern mining, and GCN. This paper focuses on the GCN-based GAD algorithm.

Due to divergent definitions of “anomaly” in academic and industrial contexts, coupled with the variability in identifying anomalous data across different scenarios, the precise characterization of what constitutes an “anomaly” proves challenging [3]. This paper abstains from delving into the root cause behind the classification of specific data as anomalous or enumerating the features that render data anomalous. Instead, our focus remains exclusively on the handling of datasets with anomaly ground-truth. Leveraging information from node features, edge features, and graph structures, we seek to infer potential label values for unlabeled nodes based on existing positive and negative labels. The objective is to simplify the model into a semi-supervised learning classification task rooted in graph neural networks, and the main task is to determine whether a node on the graph is an anomaly or not. This classification approach is also often employed in GAD tasks [5].

We propose a unified model capable of addressing the aforementioned three challenges in graphs to make full use of the information in the nodes and edges to improve the performance of the algorithm. The primary contributions of this paper include the following.

1. We analyzed the theoretical relationship between the feature convolution process of GCN and the label propagation process of LPA, demonstrating that using LPA as an auxiliary regularization term in the GCN loss function can train edge weights and thereby improve GCN performance. Building on this theoretical analysis, we introduced a learnable edge weight into GCN and trained it using LPA. This method modifies the convolution process of GCN, making full use of node labels.
2. Considering the heterogeneity of edges, we analyzed the impact of edge information on GAD tasks. Firstly, we embedded edge direction and attribute information to generate edge features. Then, we aggregated these edge features with node features to generate co-embedding. This method conducted feature extraction, fully utilizing the information on edges.
3. Based on the co-embedding, we designed separate trainable GCN weights for node features and co-embedding features, projecting them into different feature spaces, greatly enhancing the model’s generalization ability.

We conducted experiments on the publicly available DGraph [6] dataset, selecting MLP, Node2Vec [7], LPA [8], GCN [9], GAT [10], GATv2 [11], GraphSAGE [12], and UniMP [13] as baseline models, with AUC serving as the evaluation metric, which refers to the area under the ROC curve. The ROC (Receiver Operating Characteristic) curve is a graphical visualization that provides an intuitive representation of a model’s performance. As a numeric value, the AUC can directly and intuitively exhibit the goodness or poorness of a model’s performance. The results indicate that our proposed method outperforms the baselines in terms of AUC. Our model proves feasible in addressing GAD tasks, providing valuable insights for applications in scenarios with scarce data.

The remaining sections of this paper are organized as follows. Section 2 discusses related work in the field of anomaly detection. Section 3 defines the mathematical model of the problem, analyzing the theoretical connection between GCN’s convolution on node features and LPA’s propagation on node labels, providing a mathematical basis for incorporating node labels into the GCN model. Section 4 introduces our GCN-based GAD model, which is achieved through three enhancements to the GCN network. Section 5 presents experimental results on public datasets, comparing the performance with baseline models, indicating that our method exhibits the best AUC performance on datasets with extremely low proportions of anomalous nodes. Finally, in Section 6, we summarize the key contributions of this paper, as well as directions for future improvement.

## 2. Related Work

Anomaly detection algorithms typically view the task as a node classification problem, where data are categorized into normal and abnormal classes [3,4,5]. Our algorithm conceptually relates to semi-supervised methods in graph-based learning and aligns with recent advancements in applying convolutional neural networks to graph-structured data.

### 2.1. Limitations Encountered by Current Algorithms

Traditional anomaly detection methods can be divided into rule-based methods and probability-based methods. Rule-based methods aim to formalize expert knowledge rules and replicate them through computational means, while probability-based methods rely on statistical knowledge and assume that data distribution conforms to certain statistical distributions. Traditional anomaly detection methods are highly interpretable, but they need to design different detection rules for different scenarios, which requires the introduction of a large amount of expert knowledge. This method has high time and labor costs, and the generalization of the method is weak.

### 2.2. Anomaly Detection Based on Semi-Supervised Methods

Machine learning anomaly detection methods can be categorized into supervised, semi-supervised, and unsupervised learning. In cases where the number of negative samples is small, resulting in a large disparity between positive and negative sample proportions, and the patterns of negative samples are difficult to learn, supervised learning methods are not applicable. Unsupervised learning does not require considering prior knowledge and is suitable for most cases where most objects are normal. However, due to insufficient input information, unsupervised learning is prone to the curse of dimensionality, leading to low accuracy and high false-positive rates in existing patterns.

Semi-supervised learning does not rely on external interaction and automatically leverages unlabeled samples to enhance learning based on a training set composed entirely of normal data [14,15]. It allows for joint learning between a small number of labeled samples and a large number of unlabeled samples [16], leading to improved learning performance and generalization ability [17,18]. By better capturing data distribution characteristics, it is particularly suitable for scenarios with limited labeled data and expensive manual annotation. Specifically, semi-supervised learning can be categorized into inductive learning (or pure semi-supervised learning) and transductive learning [14]. In the former, the assumption is that the unlabeled samples in the training data do not represent the data to be tested, while in the latter, the unlabeled samples considered during the learning process are precisely the ones to be predicted [17].

In the mentioned methods, LPA and GNN methods are the focal points of our attention, they can be summarized in Table 1. From the table, we observe that LPA is typically associated with transductive learning, implying its inability to generalize to new nodes; instead, it solely classifies the remaining nodes in the original graph. This is because, during the model learning process, nodes with unknown labels in the original graph may also be utilized for training. In other words, certain unlabeled data obtain their label information from other unlabeled data. GNN is associated with inductive learning [19]. Additionally, GNNs can leverage node features and support graph embedding, whereas LPA cannot. LPA, however, can make full use of node label information, which GNNs may not fully exploit. Consequently, both algorithms alone may not effectively utilize the information present in the nodes of the graph. Our primary research goal is to cleverly combine these two algorithms.

LPA was initially proposed by Zhu and Ghahramani [8], and implemented using bipartite graphs. Several anomaly detection algorithms have been proposed based on LPA. Li et al. [20] used adaptive label propagation for group anomaly detection in networks. Wang et al. [21] introduced a collaborative multi-label propagation strategy, leveraging a user-item interaction graph for acceleration. Wang et al. [22] improves graph-based label propagation algorithm with group partition for fraud detection.

With GNN algorithms, numerous effective anomaly detection algorithms have also been proposed. Building upon the dynamic heterogeneous graph, Zhang et al. [23] introduced the hierarchical multi-modal fusion GNN (HMF-GNN), which was applied to the detection of health insurance claim records. This model addresses the challenge of lost neighbor information. DAGAD [24] provides a promising solution to alleviating the problem of GAD with anomalous sample scarcity and class imbalance.

However, the aforementioned methods have not been able to fully integrate the information provided by node features and labels.

### 2.3. Anomaly Detection Based on Graph Convolutional Networks

According to whether the node attributes in the graph are multivariate, graphs can be classified into homogeneous and heterogeneous graphs [25], this paper exclusively discusses homogeneous graphs. For graph data, anomalies can be considered as elements such as nodes, edges, subgraphs, etc., that differ from the majority of objects in the graph. We can categorize GAD into different types based on the properties of the graph (static graph, static attribute graph, and dynamic graph), and the nature of anomalies (node-based, edge-based, and graph-based) [4,26]. The focus of this paper is on the detection of anomalous nodes in static attribute graphs. In attribute graphs, besides the structural information between nodes, each node also encompasses attribute information [27]. These attribute details aid in uncovering hidden features of nodes, thereby enhancing the accuracy of node anomaly detection.

Our approach is primarily inspired by GCN-based algorithms. Kipf and Welling [9] introduced the concept of convolution to graph structures, proposing GCN. Building on the work of GCN, Hamilton et al. [12] improved the mean aggregation method and introduced GraphSAGE, a method for inductive representation learning, enabling algorithmic application to large-scale graphs through minibatch sampling. However, GAD algorithms constructed based on the above methods, such as GEM [28] and DualFraud [29], have not taken into consideration the benefits brought by node labels.

Other methods, like GCN-LPA [30], and UniMP [13], cleverly combine GCN with LPA. Huang et al. [19] demonstrated that this combination can avoid excessive smoothing, thereby enhancing the performance of GCN. However, the aforementioned methods have not fully utilized the information embedded in edges.

Research on combining GCN with LPA for recommender systems (RS) has proven the feasibility of this approach in graph node classification [30,31,32]. Li et al. [33] combined LPA with GNN and proposed a GAD method applicable to e-commerce shopping networks.

## 3. Problem Formulation

We start with some notation. Assume that there is a graph, directed or undirected, G=(V,E), in which $V=\left\{v_{1},\ldots ,v_{I}\right\}$ denote the sets of subjects to be detected. Each node has a *P*-dimensional feature, the *i*-th node’s feature is represented by $x_{i}$. The arrangement of all features in a row by node orders the results in the feature matrix $X\in R^{I\times P}$. $A\in {\left\{0,1\right\}}^{I\times I}$ is the binary adjacency matrix, the ij-th entry $a_{ij}\in \left\{0,1\right\}$ of *A* means whether there is an edge from $v_{i}$ to $v_{j}$, and vice versa. Number the elements of *A* from the first row to the *n*-th row as 1,2,…,n+1,n+2,…,nn−1,nn; therefore, *A* can be flattened into a two-dimensional array $A\in N^{2\times c}$, called the edge index matrix, where the first row represents the source node and the second row represents the target node. *c* is the number of non-zero elements in the matrix *A*, as well as the number of edges in the graph. Partition *A* into two triangular matrices, $A_{upper}$ and $A_{lower}$. For an undirected graph, the first c/2 elements of A, correspond to the ith non-zero element in $A_{upper}$; the last c/2 elements of A correspond to the *i*-th non-zero element in $A_{lower}$. *D* is the diagonal degree matrix, and $S=D^{-1/2}AD^{-1/2}$ is the normalized adjacency matrix.

**Figure 1:**
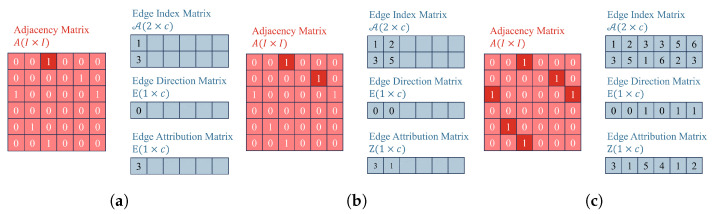
The process of mapping the adjacency matrix *A* to the edge index matrix A, as well as the construction of *E* and *Z*. (**a**) The first non-zero element in *A* is mapped to A. (**b**) The second non-zero element in *A* is mapped to A. (**c**) The remaining non-zero elements in *A* are mapped to A.

For graph *G*, each edge comprises two elements: edge direction and attribution. Matrices *E* and *Z*, are organized based on the corresponding order of edges. That is to say, *E* and *Z* shares the same topology as A. The matrix $E\in {\left\{0,1\right\}}^{1\times c}$ stores the directions of the edges, where 0 and 1 represent incoming edges and outgoing edges. The matrix $Z\in {\left\{1,\ldots ,n\right\}}^{1\times c}$ stores the attributes of the edges, where different values of element $Z_{i}$ represent different attributes of the edges. Figure 1 shows the process of mapping the adjacency matrix *A* to the edge index matrix A, as well as the construction of the edge direction matrix *E* and the edge attribution matrix *Z*. In (a), the first non-zero element in *A* is read, which is located in the first row and third column. Therefore, it represents that the corresponding edge has a source node of index 1 and a target node of index 3. Consequently, the first column of the edge index matrix A, denoted as $A_{1}$, has 1 in the row above (representing the source node) and 3 in the row below (representing the target node). For the edge direction matrix, since this element is located in the upper triangular matrix $A_{upper}$, the first element of the edge direction matrix $E_{1}$ is 0, indicating an incoming edge. The first element of the edge attribution matrix $Z_{1}$ stores attribute 3 of this edge. Following this procedure, we iterate through the other non-zero elements in *A* to construct A, *E*, and *Z*.

We can transform the directed graph *G* into an undirected graph UG through a simple symmetric operation. This involves processing the elements in matrices *A* with the transformation $a_{ij}\;\mathrm{or}\;a_{ji}=\left\{\begin{array}{cc} a_{ji}\;\mathrm{or}\;a_{ij}, & \;\mathrm{if}\;a_{ij}\;\mathrm{or}\;a_{ji}\neq 0\\ 0, & \;\mathrm{otherwise}.\; \end{array}\right.$

Consequently, *A* becomes symmetric matrices.

In GAD tasks, the node set *V* is divided into a disjoint set of unlabeled nodes and labeled nodes, while only the first *m* nodes (m≪n) have labels from a label set L={1,2}. Here, 1 represents normal nodes, and 2 represents anomalous nodes. The label is stored by a one-hot-encoding matrix $Y={\left[y_{1},\ldots ,y_{n}\right]}^{T}$.

Finally, *V* is divided into three sets in proportion: training set $V_{train}$, validation set $V_{valid}$, and test set $V_{test}$. Our objective is to learn the features of nodes with labels, propagate their labels, and then infer the labels of the remaining nodes, given *G*, *X*, *Y*. Training is performed on $V_{train}$, validation is conducted on $V_{valid}$, and testing is carried out on $V_{test}$.

Table 2 summarizes the symbols used in this paper along with their corresponding meanings.

### 3.1. Label Propagation Algorithm

LPA is based on the assumption that two connected nodes are correlated because of the homophily and influence. LPA utilizes node labels as its input.

We construct a one-hot-encoding label matrix $Y^{\left(k\right)}={\left[y_{1}^{\left(k\right)},\ldots ,y_{n}^{\left(k\right)}\right]}^{T}$ to store labels. The *i*-th row $y_{i}^{(k)T}$ stores the predicted label of node $y_{i}$ after *k* times label propagation. The label matrix $Y^{\left(0\right)}$ is initialized with the rule $y_{i}^{\left(0\right)}=\left\{\begin{array}{cc} y_{i} & i\leq m,\;\mathrm{labeled}\;\mathrm{nodes}\;\\ zero-vector & m<i\leq n,\;\mathrm{unlabeled}\;\mathrm{nodes}\; \end{array}\right.$. In the label propagation process during the *k*-th iteration, all nodes propagate their labels to neighboring nodes with Equation (1). From the perspective of nodes, node $v_{i}$ will choose the community of its neighbor with the highest membership count as its new community:

(1) $$Y^{\left(k+1\right)}=D^{-1}AY^{\left(k\right)}.$$

Labels are propagated from each other nodes through a normalized adjacency matrix $D^{-1}A$. Subsequently, all nodes are reset back to their initial labels $y_{i}^{\left(0\right)}$, before proceeding to the (k+1)-th iteration:

(2) $$y_{i}^{\left(k+1\right)}=y_{i}^{\left(0\right)},\forall i\leq m.$$

**Figure 2:**
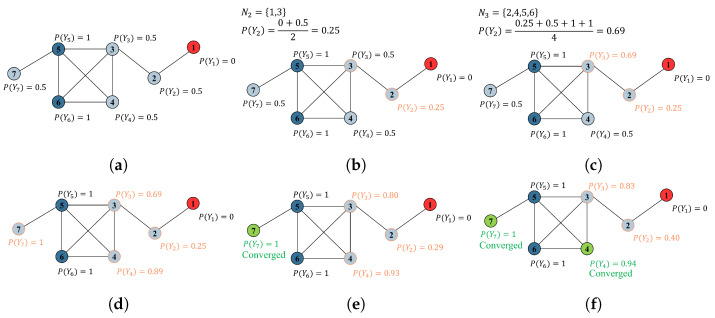
Label propagation process in three iterations: a demo. In the first iteration, the algorithm starts computation from the first unlabeled node (i.e., node 2). For node 2, $N_{2}=\left\{1,3\right\}$. Here, $N_{i}$ represents the neighboring nodes of node i. The next unlabeled node is then computed (i.e., node 3), using the updated value of node 2. After the first iteration, all nodes are reset back to their initial labels according to Equation (2). After multiple iterations (three iterations are shown here), some node values will approach convergence, representing the final label probabilities for nodes. (**a**) Initial state. (**b**) Update for node 2 in the 1st iteration. (**c**) Update for node 3 in the 1st iteration. (**d**) After a round of updates for all unlabeled nodes (iteration 1). (**e**) After iteration 2. (**f**) After iteration 3.

LPA is an iterative computation process and does not guarantee convergence. Approximately five iterations are often sufficient to achieve convergence [8], striking a balance between accuracy and performance. This will be the chosen number of iterations in our study. Consequently, for node *v*, the probability of category *l* is calculated as:

(3) $$P\left(Y_{v}=c\right)=\frac{1}{\sum_{e(v,u)}A_{v,u}}\sum_{e(v,u)}A_{v,u}P\left(Y_{u}=c\right),$$

where e(v,u)∈E represents the edge from *v* to its neighbor *u*. Figure 2 illustrates an LPA process executed on an unweighted, undirected graph.

In case of an undirected graph, the final representation of node *i* is the weighted average of its neighboring nodes j∈N(i), represented by $y_{i}^{\infty }=\sum_{j\in N(i)}a_{ij}y_{j}^{\infty }$.

### 3.2. Graph Convolutional Neural Networks

GCN [9] is one of the most popular GAD models based on the Laplacian smoothing assumption. GCN utilizes node features as its input. In general, GCN employs a sum aggregator as an aggregation function, the discussions in this paper are based on it. We begin our derivation from the spatial representation of GCN. (The connection between LPA and GCN is observed in their spatial representations, both of which have the form of AHΘ. The original derivation of GCN starts from the spectral domain of graph convolution, and the spatial representation of GCN is an approximation using Weisfeiler-Lehman on the spectral domain of graph convolution. However, for LPA, the original paper directly provides a spatial representation without a spectral domain expression. Reference [34] introduces energy to represent information propagation and then considers energy Laplacian, but its form differs significantly from the spectral domain expression of GCN.)

The feature propagation scheme of GCN in layer *l* can be formulaed as:

(4) $$H^{\left(l+1\right)}=\sigma \left(\tilde{S}H^{\left(l\right)}\Theta^{\left(l\right)}\right)$$

where σ is an non-linear activation function, $\Theta^{\left(l\right)}$ denotes the GCN trainable weight in the *l*-th layer, and $H^{\left(l\right)}$ is the *l*-th layer representations of nodes with $H^{\left(l\right)}=X$ for the input layer. To avoid neglecting the node’s own features, self-loops are added when updating the node itself. This involves combining the node’s own features with the features of its neighbors during the node update process. The normalized adjacency matrix will be $\tilde{S}={\tilde{D}}^{-\frac{1}{2}}\tilde{A}{\tilde{D}}^{-\frac{1}{2}}$, where $\tilde{A}=A+I$, and $\tilde{D}$ is the diagonal degree matrix for $\tilde{A}$ with entries ${\tilde{D}}_{ii}=\sum_{j}{\tilde{A}}_{ij}$. Of particular note is the adjacency matrix $\tilde{S}$, which here remains fixed, and depends on the topological structure of the constructed graph. Meanwhile, the weight Θ mentioned is the trainable weight of GCN, which is different from our learnable edge weight.

By stacking GCN, features for nodes in an *l*-dimensional space can be obtained. In GAD tasks, the final layer of GCN is set to an output dimension of 2, allowing for the classification of all nodes into normal/abnormal binary categories. Generally, stacking two layers of GCN already yields satisfactory results [9], and this will be the number of GCN layers used in this paper. At this point, the features of nodes after two layers of GCN are represented as:

(5) $$H^{\left(2\right)}=\sigma \left(\tilde{S}\sigma \left(\tilde{S}X\Theta^{\left(0\right)}\right)\Theta^{\left(1\right)}\right)$$

Similar to LPA, in the case of an undirected graph and sum aggregator, the final representation of a node *i* is the weighted sum of its neighboring nodes j∈N(i), represented by $h_{i}^{\infty }=\sum_{j\in N(i)}a_{ij}x_{j}^{\infty }$.

### 3.3. Relationship between LPA and GCN

According to analysis before, on the label side *L*, LPA can propagate and aggregate node labels along edges; along feature side *V*, GCN can convolute and transform node features along edges. We can also observe that Equations (1) and 4 share similarities in their forms. In this section, we attempt to answer two questions: What is the relationship between the label propagation of LPA and the feature convolution of GCN? Why can LPA serve as edge weights for training GCN?

Consider two nodes $v_{1}$ and $v_{2}$ in a graph; $v_{1}$ is unlabeled while $v_{2}$ is labeled. We study the relationship between LPA and GCN from the perspective of label influence and feature influence. Especially, if the initial label (feature) of $v_{2}$ changes, how does the output label (feature) of $v_{1}$ change. The label (feature) influence can be measured by the gradient (Jacobian) of the output label (feature) of $v_{1}$ with respect to the initial label (feature) of $v_{2}$ [35,36]. The label influence of $v_{2}$ on $v_{1}$ after *k* iterations of LPA is:

(6) $$I_{l}\left(v_{1},v_{1};k\right)=\frac{\partial y_{1}^{\left(k\right)}}{\partial y_{1}}.$$

Due to the iterative nature of the LPA algorithm, the influence of $x_{2}$ on $x_{1}$ accumulates, and can eventually be expressed as:

(7) $$I_{l}\left(v_{1},v_{2};k\right)=\sum_{j=0}^{k-1}\frac{\partial y_{1}^{\left(k\right)}}{\partial y_{2}^{\left(j\right)}}.$$

On the other hand, denote $x_{1}^{\left(k\right)}$ as the *k*-th layer embedding of $v_{1}$ in GCN, and $x_{2}$ represents the initial embedding of $v_{2}$. The feature influence of $v_{2}$ on $v_{1}$ after *k*-layers of GCN is the L1-norm of the expected Jacobian matrix [30], as:

(8) $$I_{f}\left(v_{1},v_{2};k\right)={∥E_{\Theta^{\left(\cdot \right)}}\left[\frac{\partial x_{1}^{\left(k\right)}}{\partial x_{2}}\right]∥}_{1},$$

where the expectation is taken under the transformation matrix $\Theta^{\left(\cdot \right)}$. The feature influence should be normalized as:

(9) $${\tilde{I}}_{f}\left(v_{1},v_{2};k\right)=\frac{I_{f}\left(v_{a},v_{b};k\right)}{\sum_{v_{i}\in V}I_{f}\left(v_{1},v_{i};k\right)}.$$

Assume that the ReLU function serves as the activation function σ of GCN, and β denotes the fraction of unlabeled nodes. Consequently, the relationship between the label propagation of LPA and the feature convolution of GCN can be concluded, which can be expressed as:

(10) $$E_{\Theta^{\left(\cdot \right)}}\left[I_{l}\left(v_{1},v_{2};k\right)\right]=\sum_{j=0}^{k-1}\beta^{j}{\tilde{I}}_{f}\left(v_{1},v_{2};j\right).$$

The details of the proof for Equation (10) can be found in Reference [30]. With this, the first question posed in this section is answered: the relationship between label propagation and feature convolution is that if the label of node $v_{2}$ has a sufficiently large influence on the label of node $v_{1}$, then the initial features of node $v_{2}$ will also have an equally large impact on the output features of node $v_{1}$. In other words, the influence of all nodes labeled as *c* on the label of node $v_{1}$, i.e.,

(11) $$\sum_{\forall v_{2},\;\mathrm{label}\;\mathrm{of}\;v_{2}\;\mathrm{is}\;c}I_{l}\left(v_{1},v_{2};k\right)$$

is proportional to the probability of node $v_{1}$ being classified as *c* by LPA as Equation (3), i.e.,

(12) $$P\left({\hat{y_{1}}}^{lpa}=c\right).$$

From the above derivation, we can conclude that label influence can serve as a substitute for feature influence, which is the answer to the second question. This provides a theoretical basis for us to use LPA to assist in training edge weights for GCN and to establish a GCN model where edge weights are learnable.

## 4. Our Unified GCN-Based GAD Model

**Figure 3:**
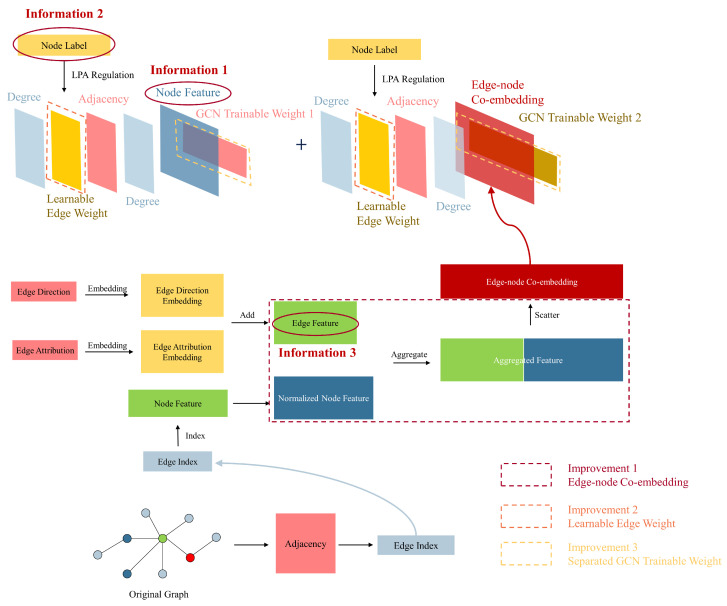
Overall architecture of our unified Graph Convolutional Network (GCN)-based Graph Anomaly Detection (GAD) model.

**Figure 4:**
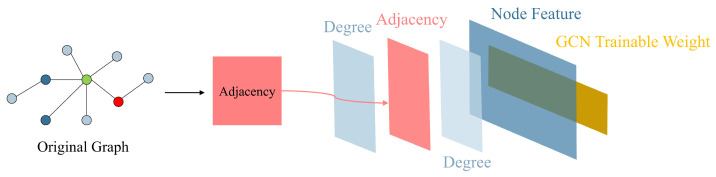
Architecture of classical Graph Convolutional Network (GCN).

This section will introduce our unified GCN-based GAD model, primarily consisting of three clever enhancements built upon the GCN network: learnable edge weights, edge-node co-embedding, and separate trainable GCN weight. Learnable edge weights introduce node label information through LPA, edge-node co-embedding incorporates information from edges, and separate trainable GCN weights enhance the model’s expressive ability. We aim to leverage the improvements mentioned above to fully utilize node feature information, node label information, and edge information in the GAD graph, thereby enhancing the algorithm’s performance in scenarios with extremely low proportions of anomalous data. Figure 3 illustrates the overall architecture of our unified GCN-based GAD model. In contrast, Figure 4 illustrates the structure of a classical GCN network.

### 4.1. Learnable Edge Weight

To ensure narrative coherence, we first introduce the concept of learnable edge weights.

**Figure 5:**
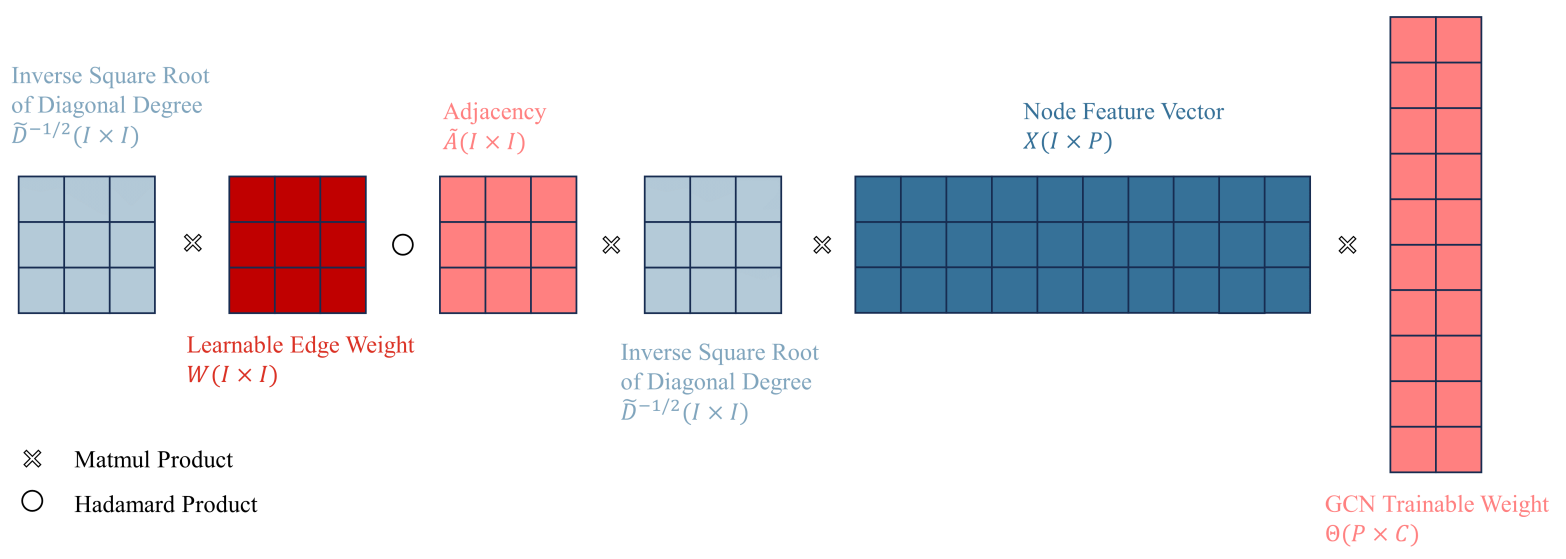
First improvement: Introducing node label information by adding learnable edge weights (only one layer of Graph Convolutional Network (GCN) is displayed).

Building upon traditional GCN networks, as in Equation (4), a learnable edge weight parameter $W^{\left(l\right)}$ is introduced. With this addition, the GCN network can be rewritten as:

(13) $$H^{\left(l+1\right)}=\sigma \left({\tilde{D}}^{-\frac{1}{2}}W^{\left(l\right)}\circ \tilde{A}{\tilde{D}}^{-\frac{1}{2}}H^{\left(l\right)}\Theta^{\left(l\right)}\right),$$

where $W_{\left(l\right)}$ represents the edge weight parameter for layer *l*, ∘ is the symbol of the Hadamard product. It is important to note that the matrix $\tilde{A}$ in the equation is constant and solely dependent on the adjacency relations of the input nodes, with its values remaining unchanged throughout the training process. Θ represents the weights of the graph neural network (not our learnable edge weight). Figure 5 illustrates our first improvement, which is introducing node label information by adding learnable edge weights. The number of columns in the GCN trainable weight layer, denoted as C, is variable. It is set to 2 in the final GCN layer (meeting the binary classification requirements of our GAD task), while in other layers, it is set to the number of hidden units.

The next question becomes how to train the weight matrix $W\circ \tilde{A}$ to optimize its performance. Combining Equations (11) and (12), we can conclude that when the loss function of LPA $L_{LPA}\left(W\circ \tilde{A}\right)$ is minimized, the optimal edge weight $\hat{W\circ \tilde{A}}$ is achieved, as:

(14) $$\hat{W\circ \tilde{A}}=\underset{W\circ \tilde{A}}{\arg\min}L_{LPA}\left(W\circ \tilde{A}\right).$$

Cross entropy can measure the distance between predicted values ${\hat{y}}_{1}^{lpa}$ and ground truth $y_{1}$. Therefore, the above expression can be rewritten as:

(15) $$\hat{W\circ \tilde{A}}=\underset{W\circ \tilde{A}}{\arg\min}\frac{1}{\mathrm{number}\;\mathrm{of}\;\mathrm{labeled}\;\mathrm{nodes}}\sum_{\mathrm{labeled}\;\mathrm{nodes}\;v_{1}}J\left({\hat{y}}_{1}^{lpa},y_{1}\right),$$

where *J* is the cross entropy loss.

As a GAD task, as analyzed in Section 1, fraudulent activities exhibit correlations and organizational characteristics. Fraudulent groups often have close connections, and their behaviors show certain similarities. This implies that neighboring nodes in the graph often have the same labels, and their feature vectors are also similar (as the node feature vectors in the GAD task are derived from real user behavior). Given that neighboring nodes have similar labels and feature vectors, cross entropy can effectively encourage adjacent nodes to be similar, learning an effective mapping from feature vectors to labels. Therefore, the loss function for traditional GCN is defined as follows: cross entropy is also used to compute the distance, and the loss of predicted labels by GCN $J\left({\hat{y}}_{1}^{gcn},y_{1}\right)$ minimized to learn the optimal transformation matrix $\Theta^{*}$ in the GCN:

(16) $$\Theta^{*}=\underset{\Theta }{\arg\min}\frac{1}{\mathrm{number}\;\mathrm{of}\;\mathrm{labeled}\;\mathrm{nodes}}\sum_{\mathrm{labeled}\;\mathrm{nodes}\;v_{1}}J\left({\hat{y}}_{1}^{gcn},y_{1}\right).$$

The final question is how to determine the loss function for the unified model. For a graph semi-supervised learning task, we can utilize both node features and graph structure information. Therefore, we can optimize both loss functions together and incorporate L2 regularization [9]:

(17) $$L=L_{\mathrm{labeled}}+\lambda L_{\mathrm{reg}}+\gamma {∥\omega ∥}_{2}^{2},$$

where $L_{\mathrm{labeled}}$ represents the loss function based on labeled data, $L_{\mathrm{reg}}$ represents the loss function based on graph structure information, ${∥\omega ∥}_{2}^{2}$ is the L2 regularizer, and λ and γ are hyperparameters that respectively adjust the relative importance of $L_{\mathrm{reg}}$ and ${∥\omega ∥}_{2}^{2}$. Data loss $L_{\mathrm{labeled}}$ considers the distance between the predicted values and the ground truth, which is the supervised loss. As a regularization penalty term, $L_{\mathrm{reg}}$ only considers the loss induced by the weight coefficients Θ.

As per Section 3.3, it is evident that LPA has the ability to replace GCN in training edge weights. Therefore, the loss of LPA (Equation (15)) can replace the regularization penalty term $L_{\mathrm{reg}}$ in Equation (17) for training, thereby updating *W* (Equation (13)), which is the proposed learnable edge weight. The data loss in Equation (17) is composed of the loss of traditional GCN (Equation (16)). Ultimately, the loss function can be written as:

(18) $$L=\frac{1}{m}\sum_{\mathrm{labeled}\;\mathrm{nodes}}J\left({\hat{y}}^{gcn},y\right)+\lambda \left(\frac{1}{m}\sum_{\mathrm{labeled}\;\mathrm{nodes}}J\left({\hat{y}}^{lpa},y\right)\right)+\gamma {∥\omega ∥}_{2}^{2},$$

where *m* is the number of labeled nodes in the graph. The first term corresponds to the segment of GCN that learns the edge weights *W* and the trainable matrix Θ. Meanwhile, the second term corresponds to the label propagation segment, which can be seen as imposing constraints on the edge weights *W*. The second term can be viewed as a regularization on *W* to assist GCN in learning edge weights.

The above process is implemented using Algorithm 1. First, we initialize a trainable edge weight matrix $W^{\left(0\right)}$ as a matrix of ones (line 2). Then, we utilize GCN to perform feature convolution, setting the output dimension of the last GCN layer to 2 for binary classification, obtaining the GCN’s label predictions ${\hat{y}}^{gcn}$ (lines 4–8). The computation of LPA is similar to GCN, resulting in the LPA’s label predictions ${\hat{y}}^{lpa}$ (lines 9–12). Subsequently, the cross-entropy losses between ${\hat{y}}^{gcn}$ and ground truth *y* are calculated, as well as ${\hat{y}}^{gcn}$ and *y*, to obtain the total loss L (line 13). Finally, the GCN training weights Θ and the learnable edge weight *W* are updated using gradient descent.
**Algorithm 1** Learnable edge weight.**Input:** 
Graph G(V,E); adjacency matrix *A*; label matrix *Y*; degree matrix *D*;number of GCN layers *L*; number of LPA iterations *K*;square matrix *W*, with the size equal to the number of edges in *E*, where all elements are ones.**Output:** 
Predicted abnormal nodes out.1:**for** i=1 number of edges in *E* **do**2:  Set $W^{\left(0\right)}\left[i\right]$ to 13:**end for**4:**for** l=1 *L* **do**5:  $H^{\left(1\right)}\leftarrow X$6:  $H^{\left(l\right)}\leftarrow$ GCN convolution with Equation (4);7:**end for**8:${\hat{y}}^{gcn}\leftarrow$ Set output dimension of GCN to 2;9:**for** k=1 *K* **do**10:  $Y^{\left(1\right)}\leftarrow Y$11:  ${\hat{y}}^{lpa}\leftarrow$ LPA propagation with Equations (1) and (2);12:**end for**13:L← Calculated loss with Equation (18);14:Backpropagation;15:Update Θ and *W* 16:**return** out

### 4.2. Co-Embedding of Edge and Node Features

This section will focus on constructing edge embeddings and aggregating them with node embeddings. The amount of information contained in *H* directly determines the feature influence of a node on its neighboring nodes (as described in Equation (9)). We start by considering increasing the information contained in *H*. By adding the embeddings of edge direction and properties and generating edge features, we introduce a method to aggregate node features and edge features into a co-embedding feature.

As discussed in Section 3, each edge consists of two elements: edge direction and attribution. Reiterating the definition provided, matrix $E\in {\left\{0,1\right\}}^{1\times c}$ stores the direction of the edges, and matrix $Z\in {\left\{1,\ldots ,n\right\}}^{1\times c}$ stores the attribution of the edges.

Once the edge direction and edge attributes are defined as matrices, they can be aggregated using an additive approach for propagation [37]. Although this simple aggregation calculation is computationally efficient, it fails to capture dependencies between different relationships and interactions between node and edge features [38].

To ensure uniform dimensions between node feature matrices and edge feature matrices, while also reflecting the relationship between node and edge features, incorporating edge features into node features is needed. This involves performing aggregation operations on both node features and edge features.

First, we map the high-dimensional discrete features *E* and *Z* to continuous low-dimensional vector spaces, obtaining dense vectors, which enables the model to better handle information from *E* and *Z*. The embedding layer maps each node with values (both *e* and *z*) to a *d*-dimensional dense vector representation, denoted as $E_{emb}$ and $Z_{emb}$. The calculation process of the embedding layer can be expressed as:

(19) $$E\left[E\right]=E_{emb},$$

(20) $$E\left[Z\right]=Z_{emb},$$

where $E\in R^{v\times d}$ is the weight matrix of the embedding layer. For the edge direction matrix and edge attribute matrix, *v* is 2 and *n*, respectively, representing the dimensionality of the input space for the embedding layer. Now, we have obtained matrices $E_{emb}\in R^{c\times d}$ and $Z_{emb}\in R^{c\times d}$ after the embedding operation, and perform matrix addition to obtain the edge features:

(21) $$F=E_{emb}+Z_{emb},$$

where $F\in R^{c\times d}$.

Secondly, we expand the node feature matrix *X* to $\hat{X}$. We index the node feature vector based on the elements stored in the first row $A_{1}$ (source node) of the edge index matrix A. The elements in A correspond to the row numbers in *X*. Since the same element value may appear multiple times in A (because the out-degree of a specific node may be greater than one, i.e., it may act as a source node multiple times), there will be duplicate rows in the extended node feature matrix $\hat{X}$. Each row in the extended node feature matrix $\hat{X}$ is a copy of the corresponding row in the node feature matrix *X*. For example, if element 2 appears twice in A, then the row corresponding to $X_{2}$ will also appear twice in $\hat{X}$. Ultimately, $X\in R^{I\times P}$ is expanded to $\hat{X}\in R^{c\times P}$, where *c* is the number of edges in the graph and *P* is the dimensionality of the node features.

Thirdly, concatenate *F* with $\hat{X}$ to obtain the aggregated feature matrix $\hat{F}$, where *c* is the number of edges in the graph, and P+d is the sum of the dimensions of the edge embedding and the extended node feature.

**Figure 6:**
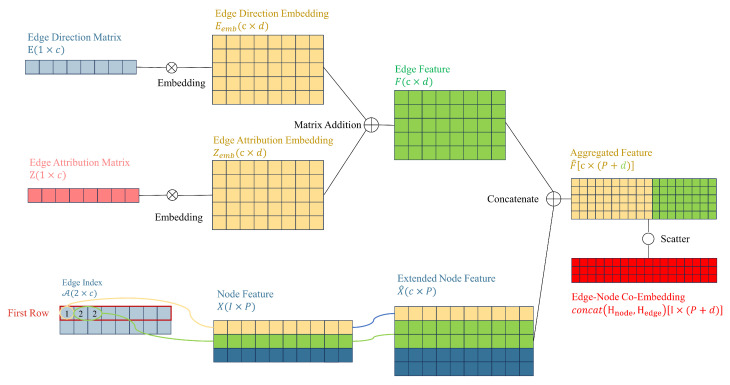
Second improvement: Introducing information from edges through edge-node co-embedding.

Finally, $\hat{F}$ is scattered to transform edge features into node features. $\hat{F}$ is set as the input tensor, and scatter indices are specified at target node positions from the second row of A. We specify the output tensor size as the number of nodes (the dimension of adjacency matrix *A*, denoted by *I*), and aggregate the data using the sum method. Ultimately, we obtain the co-embedding matrix $\mathrm{concat}\left(H_{node},H_{edge}\right)\in R^{I\times \left(P+d\right)}$ of node features and edge features. Figure 6 illustrates the process of generating co-embeddings by embedding edge direction and edge attributes, combining them with extended node features to generate aggregated features, and then scattering to obtain edge-node co-embeddings.

### 4.3. Separate Trainable GCN Weights

In this section, we design separate trainable GCN weights for node features and co-embedding features. The term “separate trainable GCN weights” refers to splitting the original trainable weight Θ in GCN into two weights, $\Theta_{1}$ and $\Theta_{2}$. These weights are used to transform node embeddings and co-embeddings in their respective feature spaces before combining them through weighted summation.

In theory, our method extracts high-dimensional information from the neighborhood of graph nodes by dimensionality reduction into dense vector embeddings. These embeddings can be provided to downstream machine learning systems to accomplish the GAD task. In graph analysis tasks, edge embeddings have been proven to be highly useful as input features for GAD [39].

Our discussion still starts with the traditional GCN. For the sake of simplicity in exposition, we rewrite Equation (4) in a more concise form:

(22) $$H_{node}^{\prime }=H_{node}\Theta .$$

In the above equation, the activation function σ and normalized adjacency matrix $\tilde{S}$ are omitted, and only one layer of GCN is considered, which does not affect the construction of our aggregation function. The equation can be seen as the transformation of node embeddings *H* to obtain new embeddings $H^{\prime }$ through trainable weight Θ, where $H_{node}^{\left(0\right)}=X$.

To cleverly combine node embedding $H_{node}$ and co-embedding $\mathrm{concat}(H_{node},H_{edge})$, we designed different Θ for $H_{node}$ and $\mathrm{concat}(H_{node},H_{edge})$, projecting them into different feature spaces. This helps enhance the model’s expressive ability, which can be written as follows:

(23) $$H^{\prime }=H_{node}\Theta_{1}+\phi \cdot \mathrm{concat}\left(H_{node},H_{edge}\right)\Theta_{2},$$

here, *W* is the learnable edge weight discussed in Section 4.1, which is incorporated into the edge embedding network, ϕ is a hyperparameter that adjusts the relative importance.

**Figure 7:**
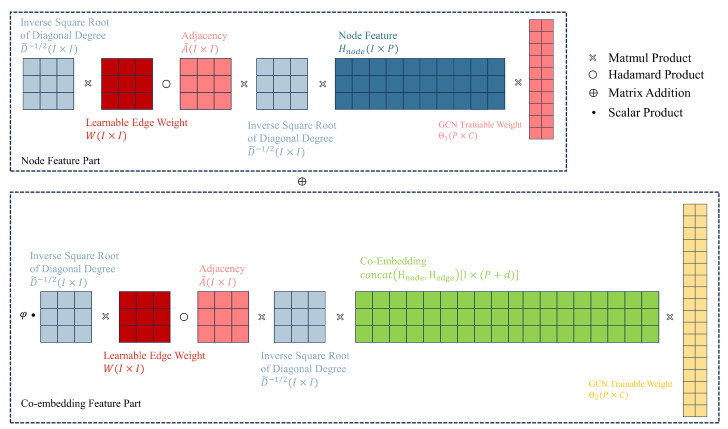
Third improvement: Designing separate Graph Convolutional Network (GCN) trainable weights to enhance the model’s expressive power (only one layer of GCN is displayed).

By substituting the co-embeddings into Equation (23), $\Theta_{1}$ and $\Theta_{2}$ can be trained separately. Figure 7 illustrates our third improvement, which is designing separate GCN trainable weights to enhance the model’s expressive power, and Algorithm 2 outlines the process of our co-embedding and separate training algorithm. First, we perform embedding operations separately on the directional and attribute information of the edges (lines 1–2). Then, we add the obtained embedding matrices to obtain the edge feature matrix (line 3). We redistribute the node feature matrix to obtain the extended node feature matrix (line 4). Next, we aggregate the edge feature matrix and the node feature matrix (line 5), and obtain the edge-node co-embedding through scatter operations (line 6). Finally, we project node features and edge-node co-embedding into different spaces using different GCN trainable weights (line 7).
**Algorithm 2** Node-edge co-embedding and separate training.**Input:** 
Graph *G*; edge direction *E*; edge attribution *Z*; edge index A; node feature *X*.**Output:** 
Predicted Abnormal Nodes out.1:$E_{emb}\leftarrow$ Embed edge direction with Equation (19);2:$Z_{emb}\leftarrow$ Embed edge attribution with Equation (20);3:$F\leftarrow E_{emb}+Z_{emb}$;4:$\hat{X}\leftarrow$ Redistribute the rows of *X* according to $A_{1}$;5:$\hat{F}\leftarrow concat\left(F,\hat{X}\right)$;6:$concat\left(H_{node},H_{edge}\right)\leftarrow scatter\left(\hat{F}\right)$;7:Convolute with Equation (23) using Algorithm 1 8:**return** out

## 5. Experiments

### 5.1. Datasets

We selected the publicly available dataset, DGraph [6], a real-world GAD graph in the finance domain, as our test sample. This dataset comprises a directed, unweighted graph that represents a social network among users. In this graph, each node represents a user, and the node features, derived from basic personal profiles, consist of a 17-dimensional vector. Each dimension corresponds to a different element of the individual’s profile, primarily including the following aspects: (1) basic personal information, such as age, gender, and location; (2) past borrowing behavior, including agreed-upon repayment due dates and actual repayment dates; (3) emergency contact-related information, such as the existence of emergency contacts. An edge from one user to another signifies a different type of connection, with a total of 12 distinct types of connections. It is noteworthy that DGraph is highly sparse, containing approximately 3 million nodes and 4 million edges. The number of nodes in DGraph is 17.1 times that of Elliptic [40], making it the largest publicly available dataset in the GAD domain to date.

As a financial dataset, DGraph originates from real-world borrowing and lending behaviors. The determination of whether a node is classified as normal or anomalous depends on its borrowing and lending activities, summarized briefly as follows. All labeled nodes in DGraph have engaged in borrowing or lending at least once. Nodes that are registered but have not engaged in borrowing or lending are classified as unlabeled nodes. Among the labeled nodes, those classified as anomalous are users who have failed to repay their loans for a considerable period after the loan’s due date, disregarding repeated reminders from the platform. According to the aforementioned criteria, 15,509 users are classified as anomalous. Among these nodes, normal users account for 22.07% (nodes labeled as 0), while abnormal users account for 0.26%. The ratio of positive to negative samples is approximately 85:1, indicating a significant class imbalance in the dataset. The nodes are randomly split into training/validation/test sets with a ratio of 70:15:15. Table 3 lists the key parameters of DGraph.

Table 4 summarizes the comparison of parameters between DGraph and mainstream anomaly detection datasets. Compared to other datasets, DGraph is closer to real-world applications because it has a minimum percentage of anomalous nodes and a diluted network, which is a common phenomenon in real GAD task. At the same time, the label data proportion in DGraph is also very low. Compared to DGraph, YelpChi is constructed using a Yelp Review Filter [41], making it not the real ground truth. The ratio of edges to nodes (in other words, the degree of the nodes) in the Amazon dataset is approximately 368:1, creating a graph that is too dense to be common in the GAD field. Elliptic is the closest to our scenario, but it treats edges as homogeneous, meaning that edges do not have attributes. In real GAD scenarios, however, the connections between users are diverse. Excessive features also reduce the usability of Elliptic. This is the reason why DGraph was chosen as the dataset for this study.

It is important to note that, for privacy protection purposes, the features of the nodes in DGraph are anonymously provided and not ordered by importance. This anonymity of features prevents this paper from visualizing the dataset in the same manner as other datasets, where the first two features are typically chosen for the x-axis and y-axis in plots. The anonymous provision of features also limits our ability to further explain which features contribute more significantly to node anomalies. (The DGraph provider anonymizes users by removing personal identifiers and randomizing transaction details, making it impossible to trace back to user IDs. Furthermore, since user features are not unique, users cannot be identified from the dataset, ensuring DGraph’s anonymity.)

**Figure 8:**
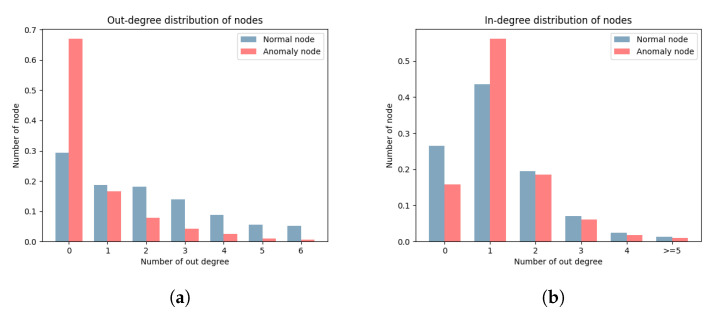
Distribution of node out-degrees and in-degrees. (**a**) Out-degree distribution of nodes. (**b**) In-degree distribution of nodes.

Figure 8 lists the distribution of node out-degrees and in-degrees. There is a significant difference in the distributions of out-degrees and in-degrees for the two types of nodes. According to the definition of DGraph, each node represents a user. The out-degree represents the completeness of information for a user, where the higher out-degree indicates higher information completeness. The in-degree represents the trust relationship of a user, where a higher in-degree indicates higher trust from other users. The out-degree of a node is determined by the user, while the in-degree is not. The significant difference between node out-degrees and in-degrees provides a basis for our edge direction embedding. The edge direction treats the out-degree and in-degree of nodes differently, accurately distinguishing between the two types of nodes.

Background nodes are an important attribute of the DGraph dataset. [6] These nodes refer to entities that lack borrowing and lending activities, are not the target of anomaly detection, but play a crucial role in maintaining the connectivity of DGraph. Throughout our experiments, we adhere to the following assumption: retain all background nodes but only perform anomaly detection on labeled nodes (normal or anomalous).

### 5.2. Baselines

We have selected eight methods as our baseline approaches, comprising one baseline method (MLP), two generic graph methods (Node2Vec and LPA), and five GCN-based methods (GCN, GAT, GATv2, GraphSAGE, and UniMP). We adopt AUC as the evaluation metric, which is extremely useful in the scenario of data imbalance where abnormal data sets are scarce while normal data sets are extremely abundant. The higher the AUC value, the better the model’s performance. For example, in DGraph, abnormal users only account for 1.16% of the dataset. Therefore, even if the model predicts all samples as normal, the accuracy would still be as high as 99%. However, it is evident that the model’s predictive performance is poor. Moreover, in GAD scenarios, the losses incurred by predicting fraudulent users as normal would be far greater than predicting normal users as fraudulent. Hence, for our GAD task, AUC is more crucial.

### 5.3. Experimental Setup

The experimental environment for all experiments remained consistent. The GPU utilized was an NVIDIA V100 (32 GB NVLink memory, single GPU with 5120 CUDA Cores), the CPU consisted of 12 Intel Xeon processors (Platinum 8163 (Skylake) @2.50 GHz), and the system had 92 GB of RAM. For each method, five runs were executed, with each run comprising 500 epochs, and the best-performing value was selected. Unless otherwise specified, all experiments are conducted on undirected graphs.

In implementing all algorithms, we utilized PyTorch Geometric. During the implementation process, we employed one trick: we replaced the storage of the matrix containing the source and target nodes of edges with a sparse adjacency matrix. This significantly accelerated computation speed, except for GAT, GATv2, and co-embedding, as they require explicit edge propagation.

All the hyper-parameters are listed below:

- Hidden layer size: 40;
- GCN layers: 2;
- LPA iteration: 5;
- Edge direction and attribution embedding size: 10;
- L2 weight: $5\times 10^{-5}$;
- Dropout rate: 0.3;
- Learning rate: 0.05.

### 5.4. Comparison with Baselines

Table 5 presents the results of the comparison between our unified model and the baseline algorithms in terms of the AUC metric. The hyperparameters used here are: LPA weight λ=1 and co-embedding weight ϕ=1. In the table, “WL” refers to the first improvement module, which is the edge weight learnable module in our model; “CE” refers to the second improvement module, which is the edge-node co-embedding module.

From Table 5, we observe that the MLP baseline, which does not utilize any graph information, achieved good results. Among the generic graph methods, Node2Vec, which only utilizes graph structure, and LPA, which only utilizes node labels, did not achieve high AUC scores. This suggests that in the GAD task on DGraph, the primary information lies within the node features. Among the GCN-based models, classical GCN and GAT performed similarly, while GraphSAGE showed significant improvement in performance. This is because GraphSAGE designs different trainable features for neighboring nodes. GATv2 improves upon GAT and exhibits noticeable performance gains. UniMP, incorporating attention mechanisms into GCN models, achieved very significant improvements.

Our edge-weight-learnable method, which is an improvement of GCN utilizing LPA to incorporate node labels, achieved higher AUC compared to traditional GCN. This demonstrates that both node features and node labels contain rich information, and LPA can act as a regularization penalty to aid training.

In terms of model training time, the general graph methods LPA and Node2Vec do not involve explicit “training” and therefore do not have training time. The MLP model, being the smallest in scale and simplest in architecture, naturally has the shortest training time. Among the methods employing the sparse matrix trick, our edge weight learnable method has the second shortest training time, only slightly longer than the classical GCN method. This is because our edge weight learnable method does not increase the model’s dimensionality; it only introduces node labels (which are also used in traditional GCN-based methods) and achieves a good trade-off between time and performance. Methods that do not use the sparse matrix trick—such as GAT, GATv2, and our co-embedding method—require significantly longer training times. This is because the edge information needs to be explicitly propagated along the edges and cannot be represented using sparse matrices to reduce computation. Additionally, extracting edge features and the growth in model size both require more time. It is worth noting that both GraphSAGE and GAT have corresponding mini-batch methods, which can reduce the size of each batch to improve speed. However, for the sake of fairness, we did not use mini-batch training in this study.

The method incorporating edge-node co-embedding and improving the convolution operator achieved significant progress in performance. This indicates that the direction and attributes of edges are helpful in enhancing the ability of GAD to detect anomalies. Additionally, designing different trainable weights for node features and co-embedding features contributes to improving algorithm expressiveness.

### 5.5. Infuence of LPA Regularization

To study the effectiveness of LPA regularization, we varied the LPA weight λ from 0 to 5 in the weight-learnable algorithm, without adding co-embedding. We observed the performance changes, as shown in Table 6.

From the table, it can be observed that when λ is non-zero (i.e., after adding learnable weight), the performance is better than before adding learnable weight. This demonstrates the effectiveness of our LPA regularization strategy. However, when λ is too large, the term $L_{\mathrm{reg}}$ in Equation (17) becomes greater than the term $L_{\mathrm{labeled}}$. In the computation of the loss, the loss function based on labeled data $L_{\mathrm{labeled}}$ is more important, as it determines the direction of gradient descent, while the regularization penalty $L_{\mathrm{reg}}$ serves as an auxiliary factor.

### 5.6. Impact of Edge and Node Co-Embedding

To investigate the influence of co-embedding, we fixed λ to 1 and varied the weight ϕ of the co-embedding term from 0 to 2, observing the performance changes. Table 7 reflects this variation. It can be seen from the table that the inclusion of edge directionality and attribute information leads to significant performance improvements, despite the fact that there are only two types of directional information and 12 types of edge attribute information. This demonstrates the effectiveness of our co-embedding method. Additionally, designing different trainable weights for node features and co-embedding features, projecting different features into different feature spaces, greatly enhances the model’s expressive ability. However, overly large ϕ values can lead to performance degradation. We believe this is because most of the information in the graph is contained within the node features, and the fundamental purpose of the GCN algorithm is to find neighboring nodes based on the similarity of node features, with edge information serving as an auxiliary.

Including reverse edges can increase the density of edges and provide more directional information, which can greatly improve performance when edge information is required for co-embedding. To demonstrate this, we fixed λ to 1 and tested the performance under the same conditions on a directed graph (i.e., without adding reverse edges). Table 7 reflects this variation. It can be observed that as the co-embedding weight ϕ increases, the performance gain from reverse edges decreases. We believe that when the co-embedding weight is low, the information contained in the graph’s topological structure is more prominent, while undirected graphs contain more topological structures. However, as the co-embedding weight increases, the directional and attribute information carried by the edges becomes more important.

To demonstrate the effectiveness of the learnable edge weight strategy, we fixed ϕ to 1 and removed the learnable edge weight on an undirected graph; the AUC decreased from 79.45% to 79.03%. This demonstrates that although the second term $\mathrm{concat}(H_{node},H_{edge})$ in Equation (23) is composed of node features $H_{node}$ and edge features $H_{edge}$ co-embedded, it is still a feature matrix aggregated based on node dimensions. Therefore, using the loss term from the LPA algorithm instead of the regularization penalty in GCN, as derived in Equation (18), still improves the algorithm’s performance.

## 6. Conclusions

This research contributes to advancing the field of graph anomaly detection, offering a robust framework for future studies and applications.

Firstly, by establishing the relationship between label propagation and feature convolution, we have shown that the LPA can function as a regularization penalty term for GCN, thereby facilitating training and enabling the learning of edge weights. Secondly, our node and edge feature co-embedding method fully utilizes the directional and attribute information inherent in the edges, increasing the spatial dimensionality of the model. Finally, we employ separate trainable GCN weights to handle node features and co-embedding features, enabling the projection of different features into separate feature spaces, greatly enhancing the model’s generalization capability.

Experimental results on the DGraph dataset have demonstrated superior AUC performance compared to baseline models, underscoring the feasibility and effectiveness of our proposed approach in tackling GAD tasks.

The method proposed in this paper currently incurs relatively high time and memory costs, requiring significant computational resources. This limitation hinders the application of the proposed method in larger-scale scenarios. Future work includes exploring techniques such as downsampling to reduce time and memory overhead while maintaining algorithm performance, making it more responsive to the rapid growth of data brought about by the Industry 4.0 era.

## Figures and Tables

**Table 1:** Comparison of semi-supervised anomaly detection methods.

	Label Propagation	Graph Neural Network
Graph Embedding	no	yes
Representation Learning	no	yes
Use Features	no	yes
Use Labels	yes	no
Transductive	yes	yes
Inductive	no	yes/no

**Table 2:** Symbols used in this paper.

Symbols	Meanings	Symbols	Meanings
*G*	graph	A	edge index matrix
*V*	node matrix	*E*	edge direction matrix
$v_{i}$	the *i*-th node	*Z*	edge attribution matrix
*Y*	label matrix	*H*	representation of nodes
$y_{i}$	the *i*-th label	Θ	GCN trainable weight
*A*	adjacency matrix	*W*	learnable edge weight
$a_{ij}$	edge from $v_{i}$ to $v_{j}$	$I_{f}$	feature influence
*D*	diagonal degree matrix	$I_{l}$	label influence
*S*	normalized adjacency matrix		

**Table 3:** Key parameters of DGraph.

Parameters	Value
# Nodes	3,700,550
# Node features	17-dimensional anonymous features
# Edges	4,300,999
Edge information	edge direction, edge attribution
Normal-to-anomaly ratio	85:1
Proportion of missing feature values	56.37%
-for normal nodes	39%
-for anomaly nodes	67%
Average node degree	1.22

**Table 4:** Comparison of parameters between DGraph and mainstream Graph Anomaly Detection (GAD) datasets.

Dateset	# Nodes	# Edges	# Labeled Nodes	# Anomalous Nodes	# Features
YelpChi [2]	45954	3846979	45954	14.50%	32
Amazon [2]	11944	4398392	11944	9.50%	25
Elliptic [40]	203769	234355	46564	9.80%	166
DGraph [6]	3700550	4300999	1225601	1.17%	17

**Table 5:** Comparison between our unified model and the baseline algorithms (with λ=1, ϕ=1). T.T. means average training time per epoch.

	Methods	AUC		T.T.
		Valid	Test	
Baseline	MLP	71.74%	72.31%	0.412 s
	LPA [8]	50.89%	50.95%	N/A
Generic Graph Methods	Node2Vec [7]	62.61%	62.92%	N/A
	GCN [9]	70.78%	72.32%	0.608 s
	GAT [10]	72.33%	73.31%	0.798 s
	GATv2 [11] (ICLR 2022)	75.26%	76.24%	0.901 s
GCN-based Methods	GraphSAGE [12]	75.48%	76.21%	0.671 s
	UniMP [13] (IJCAI 2021)	77.17%	78.38%	0.641 s
	GCN-WL	73.01%	73.82%	0.625 s
Our model	GCN-WL+CE	78.52%	79.45%	0.998 s

**Table 6:** The variation of Area Under the Curve (AUC) values due to different Label Propagation Algorithm (LPA) weights λ (without incorporating co-embedding).

λ	0 (Classical GCN)	0.01	0.1	0.5	1	5
**AUC**	72.32%	72.53%	72.99%	73.26%	73.82%	73.24%

**Table 7:** The variation of Area Under the Curve (AUC) values due to different co-embedding weights ϕ on directed and undirected graphs (with Label Propagation Algorithm (LPA) weights λ=1).

ϕ		0 (Just WL, No CE)	0.01	0.1	0.5	1	2
**AUC**	**Directed**	66.51%	72.90%	75.31%	76.77%	77.37%	76.69%
	**Undirected**	73.82%	74.54%	77.26%	79.30%	79.45%	73.23%

## Data Availability

The DGraph dataset is a publicly available dataset.

## Abbreviations

The following abbreviations are used in this manuscript:

LPA	Label Propagation Algorithm
GCN	Graph Convolutional Network
MLP	Multilayer Perceptron
GAT	Graph Attention Network
GNN	Graph Neural Network
GAD	Graph Anomaly Detection
ROC	Receiver Operating Characteristic Curve
AUC	Area Under ROC Curve

## References

[1] Dou Y., Shu K., Xia C., Yu P.S., Sun L. User Preference-aware Fake News Detection. Proceedings of the 44th International ACM SIGIR Conference on Research and Development in Information Retrieval.

[2] Dou Y., Liu Z., Sun L., Deng Y., Peng H., Yu P.S. Enhancing graph neural network-based fraud detectors against camouflaged fraudsters. Proceedings of the 29th ACM International Conference on Information & Knowledge Management.

[3] Akoglu L., Tong H., Koutra D. (2015). Graph based anomaly detection and description: A survey. Data Min. Knowl. Discov..

[4] Ma X., Wu J., Xue S., Yang J., Zhou C., Sheng Q.Z., Xiong H., Akoglu L. (2021). A comprehensive survey on graph anomaly detection with deep learning. IEEE Trans. Knowl. Data Eng..

[5] Ren J., Xia F., Lee I., Noori Hoshyar A., Aggarwal C. (2023). Graph learning for anomaly analytics: Algorithms, applications, and challenges. ACM Trans. Intell. Syst. Technol..

[6] Huang X., Yang Y., Wang Y., Wang C., Zhang Z., Xu J., Chen L., Vazirgiannis M. DGraph: A Large-Scale Financial Dataset for Graph Anomaly Detection. Proceedings of the Advances in Neural Information Processing Systems 35: Annual Conference on Neural Information Processing Systems 2022, NeurIPS 2022.

[7] Grover A., Leskovec J. node2vec: Scalable feature learning for networks. Proceedings of the 22nd ACM SIGKDD International Conference on Knowledge Discovery & Data Mining.

[8] Zhu X., Ghahramani Z. (2002). Learning from Labeled and Unlabeled Data with Label Propagation. https://citeseerx.ist.psu.edu/document?repid=rep1&type=pdf&doi=8a6a114d699824b678325766be195b0e7b564705.

[9] Kipf T.N., Welling M. Semi-Supervised Classification with Graph Convolutional Networks. Proceedings of the International Conference on Learning Representations.

[10] Veličković P., Cucurull G., Casanova A., Romero A., Liò P., Bengio Y. Graph Attention Networks. Proceedings of the International Conference on Learning Representations.

[11] Brody S., Alon U., Yahav E. How Attentive are Graph Attention Networks? In Proceedings of the International Conference on Learning Representations, Virtual, Austria, 3–7 May 2021.

[12] Hamilton W., Ying Z., Leskovec J. Inductive representation learning on large graphs. Proceedings of the Advances in Neural Information Processing Systems 30: Annual Conference on Neural Information Processing Systems 2017.

[13] Shi Y., Huang Z., Feng S., Zhong H., Wang W., Sun Y. Masked label prediction: Unified message passing model for semi-supervised classification. Proceedings of the 30th International Joint Conference on Artificial Intelligence.

[14] Zhu X., Goldberg A.B. (2022). Introduction to Semi-Supervised Learning.

[15] Chong Y., Ding Y., Yan Q., Pan S. (2020). Graph-based semi-supervised learning: A review. Neurocomputing.

[16] Van Engelen J.E., Hoos H.H. (2020). A survey on semi-supervised learning. Mach. Learn..

[17] Zhou Z.H., Zhou Z.H. (2021). Semi-supervised learning. Machine Learning.

[18] Lebichot B., Braun F., Caelen O., Saerens M. (2016). A graph-based, semi-supervised, credit card fraud detection system. Complex Networks & Their Applications V, Proceedings of the International Workshop on Complex Networks and Their Applications, Milan, Italy, 30 November–2 December 2016.

[19] Huang Q., He H., Singh A., Lim S.N., Benson A. Combining Label Propagation and Simple Models out-performs Graph Neural Networks. Proceedings of the International Conference on Learning Representations.

[20] Li Z., Chen X., Song J., Gao J. (2022). Adaptive label propagation for group anomaly detection in large-scale networks. IEEE Trans. Knowl. Data Eng..

[21] Wang H., Li Z., Huang J., Hui P., Liu W., Hu T., Chen G. Collaboration based multi-label propagation for fraud detection. Proceedings of the 29th International Conference on International Joint Conferences on Artificial Intelligence.

[22] Wang J., Guo Y., Wen X., Wang Z., Li Z., Tang M. (2020). Improving graph-based label propagation algorithm with group partition for fraud detection. Appl. Intell..

[23] Zhang J., Yang F., Lin K., Lai Y. Hierarchical Multi-Modal Fusion on Dynamic Heterogeneous Graph for Health Insurance Fraud Detection. Proceedings of the 2022 IEEE International Conference on Multimedia and Expo (ICME).

[24] Liu F., Ma X., Wu J., Yang J., Xue S., Beheshti A., Zhou C., Peng H., Sheng Q.Z., Aggarwal C.C. DAGAD: Data augmentation for graph anomaly detection. Proceedings of the IEEE International Conference on Data Mining (ICDM).

[25] Wang X., Ji H., Shi C., Wang B., Ye Y., Cui P., Yu P.S. Heterogeneous graph attention network. Proceedings of the The World Wide Web Conference.

[26] Pourhabibi T., Ong K.L., Kam B.H., Boo Y.L. (2020). Fraud detection: A systematic literature review of graph-based anomaly detection approaches. Decis. Support Syst..

[27] Ding K., Li J., Bhanushali R., Liu H. Deep anomaly detection on attributed networks. Proceedings of the SIAM International Conference on Data Mining. SIAM.

[28] Liu Z., Chen C., Yang X., Zhou J., Li X., Song L. Heterogeneous graph neural networks for malicious account detection. Proceedings of the 27th ACM International Conference on Information & Knowledge Management.

[29] Wu B., Chao K.M., Li Y. (2023). DualFraud: Dual-Target Fraud Detection and Explanation in Supply Chain Finance Across Heterogeneous Graphs. Database Systems for Advanced Applications, Proceedings of the International Conference on Database Systems for Advanced Applications, Tianjin, China, 17–20 April 2023.

[30] Wang H., Leskovec J. (2021). Combining graph convolutional neural networks and label propagation. ACM Trans. Inf. Syst. (TOIS).

[31] Ying R., He R., Chen K., Eksombatchai P., Hamilton W.L., Leskovec J. Graph convolutional neural networks for web-scale recommender systems. Proceedings of the 24th ACM SIGKDD International Conference on Knowledge Discovery & Data Mining.

[32] Zhang Y., Yuan M., Zhao C., Chen M., Liu X. (2022). Integrating label propagation with graph convolutional networks for recommendation. Neural Comput. Appl..

[33] Li Z., Wang B., Huang J., Jin Y., Xu Z., Zhang J., Gao J. (2024). A graph-powered large-scale fraud detection system. Int. J. Mach. Learn. Cybern..

[34] Wang H., Zhang F., Zhang M., Leskovec J., Zhao M., Li W., Wang Z. Knowledge-aware graph neural networks with label smoothness regularization for recommender systems. Proceedings of the 25th ACM SIGKDD International Conference on Knowledge Discovery & Data Mining.

[35] Koh P.W., Liang P. Understanding black-box predictions via influence functions. Proceedings of the International Conference on Machine Learning.

[36] Xu K., Li C., Tian Y., Sonobe T., Kawarabayashi K.I., Jegelka S. Representation learning on graphs with jumping knowledge networks. Proceedings of the International Conference on Machine Learning.

[37] Schlichtkrull M., Kipf T.N., Bloem P., Van Den Berg R., Titov I., Welling M. (2018). Modeling relational data with graph convolutional networks. Proceedings of the The Semantic Web: 15th International Conference, ESWC 2018.

[38] Jiang X., Zhu R., Li S., Ji P. (2020). Co-embedding of nodes and edges with graph neural networks. IEEE Trans. Pattern Anal. Mach. Intell..

[39] Wang D., Cui P., Zhu W. Structural deep network embedding. Proceedings of the 22nd ACM SIGKDD International Conference on Knowledge Discovery & Data Mining.

[40] Weber M., Domeniconi G., Chen J., Weidele D.K.I., Bellei C., Robinson T., Leiserson C.E. (2019). Anti-money laundering in bitcoin: Experimenting with graph convolutional networks for financial forensics. arXiv.

[41] Mukherjee A., Venkataraman V., Liu B., Glance N. What yelp fake review filter might be doing?. Proceedings of the International AAAI Conference on Web and Social Media.

